# Highly efficient cellular cloning using Ferro-core Micropallet Arrays

**DOI:** 10.1038/s41598-017-13242-1

**Published:** 2017-10-12

**Authors:** Trisha M. Westerhof, Wesley A. Cox-Muranami, Guann-Pyng Li, Mark Bachman, Hung Fan, Edward L. Nelson

**Affiliations:** 1 School of Medicine, Department of Medicine, Division of Hematology/Oncology, University of California, Irvine, United States; 2 Samueli School of Engineering, Department of Biomedical Engineering, University of California, Irvine, United States; 3 Francisco Ayala School of Biological Sciences, Department of Molecular Biology and Biochemistry, University of California, Irvine, United States; 4 NCI-designated Chao Family Comprehensive Cancer Center, Irvine, United States

## Abstract

Advancing knowledge of biological mechanisms has come to depend upon genetic manipulation of cells and organisms, relying upon cellular cloning methods that remain unchanged for decades, are labor and time intensive, often taking many months to come to fruition. Thus, there is a pressing need for more efficient processes. We have adapted a newly developed micropallet array platform, termed the “ferro-core micropallet array”, to dramatically improve and accelerate the process of isolating clonal populations of adherent cells from heterogeneous mixtures retaining the flexibility of employing a wide range of cytometric parameters for identifying colonies and cells of interest. Using transfected (retroviral oncogene or fluorescent reporter construct) rat 208 F cells, we demonstrated the capacity to isolate and expand pure populations of genetically manipulated cells via laser release and magnetic recovery of single micropallets carrying adherent microcolonies derived from single cells. This platform can be broadly applied to biological research, across the spectrum of molecular biology to cellular biology, involving fields such as cancer, developmental, and stem cell biology. The ferro-core micropallet array platform provides significant advantages over alternative sorting and cloning methods by eliminating the necessity for repetitive purification steps and increasing throughput by dramatically shortening the time to obtain clonally expanded cell colonies.

## Introduction

Biological scientists use a wide array of genetically modified cells as tools to dissect biologic mechanisms. The utility of these reagents is critically dependent upon having a pure cell population of defined phenotype. This requires the isolation and purification of clonally expanded colonies of manipulated cells from a heterogeneous population, e.g. 1) clonal cell lines with transient or stable, over- or decreased-expression of a particular molecule, or 2) lineage specific stem cell progeny. There are challenges that impact the efficiency of this process. The genetic manipulations of cells, typically involving some form of transfection, commonly result in the desired product representing a small fraction of the total population. Additionally, differential growth rates of desired versus undesired cells can lead to the desired transfected population being difficult to isolate because they may be outcompeted by the undesired population^1^. Thus, to obtain pure cultures of transfected cells for generation of cell lines or organisms, repetitive sorting and/or isolation steps are typically required. Although extensive research has been invested in the development of superior transfection methodologies and reagents^2,3^, little attention has been given to improving cell colony isolation and sorting methods.

Fluorescence-activated cell sorting (FACS) is a commonly used methodology utilized to isolate cell populations of interest. FACS involves sorting cells, in suspension, via the detection of fluorescent tags specific for intra- or extra-cellular molecules of interest^4^. Single transfected cells are commonly sorted into individual wells of a multi-well plate (96, 384, or 1,536 wells/plate) and expanded in culture to achieve pure transfected cell colonies. The limitations of FACS methodology^5^ include: 1) the cellular trauma intrinsic to the hydrodynamic forces of the methodology, often times compromising the viability of the isolated cells, 2) the necessity for cells to be in suspension (e.g. adherent cells must be released from their substrate, typically by enzymatic digestion of adhesion molecules, and maintained in suspension), 3) the requirement for a relatively large starting population for the isolation process, 4) a small, but present, background contaminating population, and 5) the expense of the sorting instrument. While this methodology has proved to be successful, many cell types cannot be expanded clonally in this setting and requires the screening of a large number of wells. Traditionally, FACS has been more effective for non- or loosely adherent cells.

For adherent cells, isolation of clonal populations has traditionally involved the use of limiting dilution or cloning rings^6,7^. The former involves serial dilution, culturing or plating dissociated cells in a ratio of one cell to three wells. This requires screening of a large number of cell culture wells for colony growth and phenotyping. The latter method involves the use of small, several mm diameter, rings that are used to encircle desired adherent cell colonies grown on a cell culture dish, to selectively harvest cells within the rings^6^. The main advantage of this method is that the selected transfected adherent cell colonies have demonstrated their ability to grow in culture. However, the isolated population of cells is rarely pure, due to the fact that adherent cells are typically motile, so that over time cells may migrate away from the colony and become incorporated into a neighboring colony. Thus, this process, a mainstay of biological labs for decades, must be repeated several times to isolate pure clonal cell populations, a laborious and time-consuming process. More efficient, less labor and time intensive, cloning methods would be of significant benefit across a wide range of biology disciplines.

Herein, we report an adaptation of a large area magnetic micropallet array^8^, the Ferro-core micropallet array (FCMPA) and the methodology for rapid, single step cell colony sorting. The basic micropallet array platform has been refined and adapted for the interrogation of adherent cells^9–11^. The original description of the micropallet array platform^11–15^ in 2006 and 2007, demonstrated the capacity of the platform to be able to isolate single cells and small colonies, using the very robust Hela cell line. Subsequently, a number of refinements have been incorporated into the platform by our group and the Allbritton group, who together originally reported the development of the micropallet array platform. Shadpour *et al*. reported the ability to identify colonies derived from murine ES cells by gross morphology using mechanical roughening of the surface, that was required for the incorporation of a gelatin substrate^16,17^ and the coating of the micropallet array with a monoclonal antibody recognizing a cell surface molecule^18^, a functionalization of the micropallet array that has been used to capture rare cells from heterogeneous mixtures^10^. We described the ability to effectively coat the micropallet array with a variety of extracellular matrix components that could be tailored to the desired adherent cell type^19^, including methodologies to limit bridging of the extracellular matrix coating between micropallets. Xu *et al*. utilized the ability of colonies to bridge between micropallets to develop a sampling methodology for the screening of growing colonies using cell lethal assays^20^. Detwiler *et al*. described methods for incorporating polystyrene as the growth surface for the micropallet arrays^21^. The methodologies for the release and recovery of desired micropallets, to this point, remained low throughput^13,14,22^. We have described the incorporation of ferromagnetic 1002 F photoresist for the fabrication of the micropallet arrays and magnetic micropallet recovery^9^. In this same report, we described the ability to image, release and recover micropallets from arrays using a single instrument, a confocal scanning microscope^9^, a process that previously utilized two separate pieces of equipment. The uniform distribution of ferromagnetic nanoparticles throughout the photoresist results in degradation of the imaging quality, even with scanning confocal imaging^9,23^. Application of the micropallet array platform to the identification and recovery of rare cell subsets, such as cancer stem cells among others, requires the use of multiple cell surface markers. Multichannel imaging has been demonstrated for two^24^, three^25^, and six channels^26^, the latter achievable only with micropallet arrays fabricated from polymer that did not contain ferromagnetic particles. The majority of work with micropallet arrays has been directed at isolation of single cells, but the platform has been applied to colony isolation, including at the time of original description^13,14^. Pai *et al*. employed large micropallets with “cup”-like features to grow and sample colonies of ES-derived stem cells^27,28^. These arrays were designed to have colonies grow across multiple micropallets and allow sampling of a portion of the colony^28^ in a manner similar to Xu *et al*.^20^. The growth from micropallet to micropallet poses challenges for purity of putative clonal cellular populations. A more complex system of interlocking matched micropallet arrays with bridging “posts” has also been described^29^ that employs the originally described collection system^13^. This platform uses virtual air walls to restrict cells to the tops of micropallets and then provides a bridging post for a continuous growth surface to a matched, non-identical, micropallet array. The cup and post adaptations of the micropallet array platform have the advantage of being able to sample colonies and use cell destructive assays for identification of colonies of interest, although the use of reporter constructs that do not impair cell viability are currently used much more frequently.

Recently an alternative to the micropallet array has been described, the microraft array. In its original description, the rafts were fabricated of polystyrene^30^. More recent iterations of this platform incorporate copolymers of polylactic acid and polyglycolic acid in Gamma-butyrolactone (GBL)^31^ in the presence or absence of ferromagnetic particles incorporated into the polymer mixture^32,33^. Both of these microraft arrays are fabricated using dip coating of a PDMS mold, which once completely processed results in PDMS physical barriers, walls, between the microrafts. Microrafts containing desired cells or colonies, are physically dislodged by piercing through the base PDMS lifting the microraft from the mold. Released microrafts are collected by magnet if containing iron nanoparticles or by gravity with inversion of the microraft array over a culture surface (plate or wells). The former has significant degradation in optical properties and the latter has lower throughput.

We have previously described the fabrication of a micropallet array for the isolation of cell colonies, containing large micropallets with magnetic properties, and incorporating a limited ferromagnetic core into each micropallet^8^, for ease of recovery, that does not have the compromised imaging capacity of iron nanoparticle doped micropallets or microrafts. In the current report, we describe refinements to enhance and prolong the adhesion of extracellular matrix components, the capacity to tailor the culture surface to different adherent cell types, to optimize cellular adherence as previously demonstrated for the micropallet array^19^. We also describe additional refinements to effectively preclude cell migration from growth surface to growth surface (enhanced cellular sequestration) during the period of microculture and provide proof of concept for the capacity of this platform to be applied to the isolation and recovery of clonal microcolonies derived from single cells. This represents a new and refined platform and methodology for the rapid isolation of clonal populations of adherent cells of defined phenotype from heterogeneous populations. This platform and methodology has the potential to significantly accelerate biological studies requiring clonal populations of cells such as genetically manipulated cells or organisms.

## Results

### Ferro-core micropallet array fabrication, micropallet release and recovery

**Figure 1:**
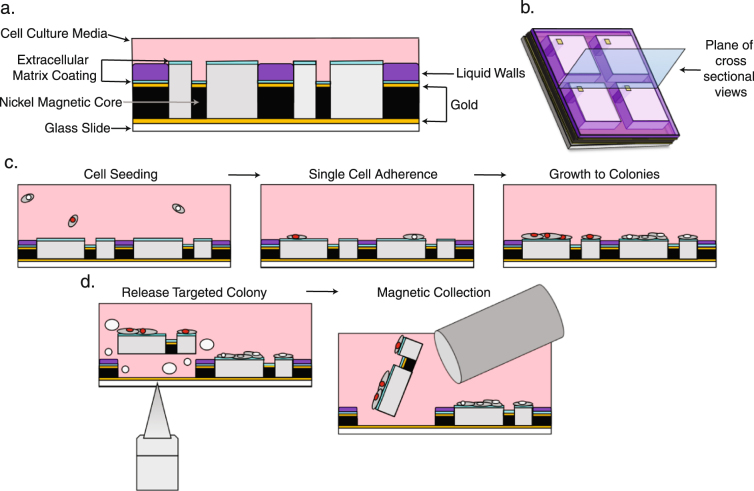
Schematic representation of FCMPAs and workflow for cell colony sorting. (**a**) FCMPs are arrayed on a glass slide coated with gold thin film, and are coated with ECM. Fluorinert™ is used to form liquid barrier walls within the channels surrounding the ECM-coated micropallets to sequester cells to the top micropallet surfaces. (**b**) A 3-D schematic of the FCMPAs demarcating the cross section (blue) that was used to generate the schematics in a,c,d. (**c**) Single cell suspensions seeded on processed FCMPAs from above were allowed to attach and clonally expand for seven days. (**d**) Individual FCMPs carrying cell colonies of interest were identified, laser released, collected using a magnetic probe, and transferred to a cell culture dish for clonal expansion.

**Figure 2:**
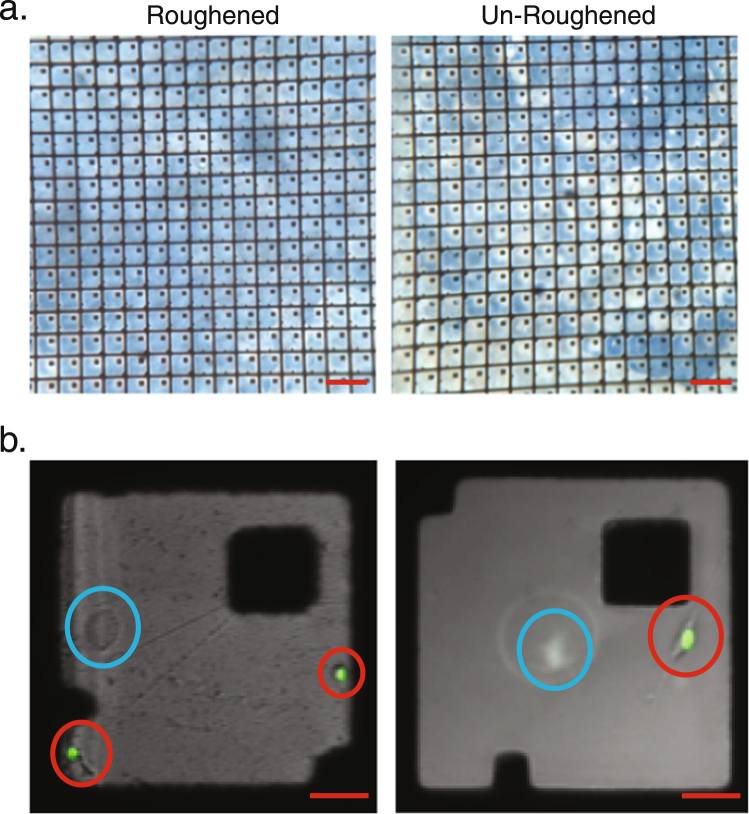
Micropallet surface roughening. (**a**) Fibronectin coatings on un-roughened and roughened FCMPAs were interrogated using colorimetric immunostaining with an anti-fibronectin antibody, horseradish peroxidase-labeled secondary antibody, and developed with colorimetric TrueBlue substrate. Representative scale bars in each phase contrast microscopic image are 500 μm. (**b**) A suspension of transfected H2b-mCherry cells pseudo-colored green (red circle) were seeded onto both un-roughened and roughened FCMPAs and allowed to adhere. Extraneous air bubbles are circled in blue. Representative scale bars in each fluorescent confocal image are 50 μm.

Herein, we report an adaptation of the large area magnetic micropallet arrays^8^, the Ferro-core micropallet array (FCMPA) and the methodology for rapid, single step cell colony sorting. The basic micropallet array is a recently described platform designed for interrogation of adherent cells^9–11^. FCMPAs were fabricated using methods previously described^8,9,34^, with seven major components for this application; A) high-precision glass slides coated with a 200 Å gold film, B) arrays of 250 µm × 250 µm × 50 µm (L × W × H) 1002 F photoresist micropallets, C) indexing notches, D) 60 µm × 60 µm square cores of electroformed ferromagnetic material, E) a second 200 Å gold film to optimize biocompatibility of the ferromagnetic core, F) deposition of one of several extracellular matrix components, and G) a method for sustained sequestration of cells to a particular micropallet (weeks). These large-scale micropallets provide a surface area available for cell adhesion ranging from 57,100 µm^2^ to 58,900 µm^2^, depending on the number of index notches^19^. The structure of the fabricated FCMPA and the workflow for post fabrication treatments, cell sequestering, micropallet release and recovery are depicted in Fig. 1. Different types of adherent cells have optimal extracellular matrix substrates for adhesion and sequestering on 1002 F micropallets^19^. We found, as have others^17^, that for large surface area micropallets, surface roughening provides better and longer lasting adhesion of the extracellular matrix component coating, Fig. 2.

**Figure 3:**
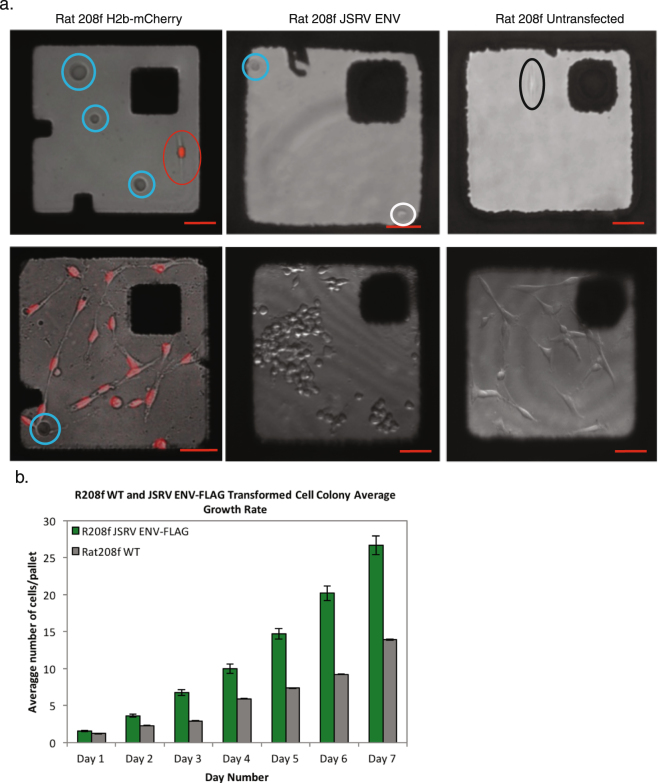
Application and growth of cells on FCMPAs. (**a**) Representative fluorescent confocal images of single cells on FCMPs (top panels), and images after seven days of growth to form microcolonies (bottom panels). Single cells are circled for rat 208 f H2b-mCherry (red), JSRV ENV (white) and untransfected wild type (black) and air bubbles were circled in blue for distinguishment. All confocal images contain 50 μm scale bars. (**b**) Growth rate of transformed JSRV ENV-FLAG expressing and wild type R208f cells, plated as single cells, grown on fibronectin coated FCMPA, with manual counts of cell numbers on individual micropallets for 7-days. An average of 95 micropallets containing JSRV ENV-FLAG R208f cells (n = 102, 77, 99, 74, 98, 114, and 100 for days 1–7 accordingly), and an average of 35 micropallets containing WT R208f cells (n = 38, 38, 40, 41, 35, 28, and 28 for days 1–7 were counted daily.

After adhesion and growth of microcolonies, the release of micropallets with adherent cell colonies was accomplished by focus of a two-photon laser at the interface of the micropallet base and thin gold layer, resulting in energy absorption, heat generation, and formation of gas micro-bubbles that released the Ferro-core micropallet (FCMP); a method distinct from that described for standard micropallet arrays^8,22^. Single FCMPs are released and collected individually to avoid simultaneously collecting more than one micropallet. Released FCMPs come to rest on the surface of the array and were collected using a rare earth neodymium magnet^8,9^. Isolated or adjacent FCMPs can be released without disturbing nearby micropallets with cellular viability exceeding 85% (Fig. 3).

To permit extended culture of cells adherent to the FCMPA and the ability to reproducibly obtain a clonal colony, while still permitting the coating of the FCMPA with one of several extracellular matrix preparations, required an alternative method to sequester cell(s) to individual micropallets. Thus, in lieu of silanization and creation of virtual air walls or physical (PDMS) walls, the space between micropallets was occupied by a very small amount (0.5 ul) of Perfluorotributylamine (PFTBA), also marketed as Fluorinert FC-43, a lipophobic and hydrophobic liquid with nearly twice the density of water, which was added after application of the extracellular membrane component coating. The sequence of the post fabrication treatments is critical. The Fluorinert compounds have been repeatedly demonstrated to be biocompatible^35–37^, while we and others^38^ have observed that the Fluorinert compounds do not support cell growth on or cellular migration across the interface surface, unless a protein layer is deposited on the interface or a surfactant is incorporated into the Fluorinert^35,39^, (Supp Figure 2).

### Cell clone sorting, recovery and expansion

**Figure 4:**
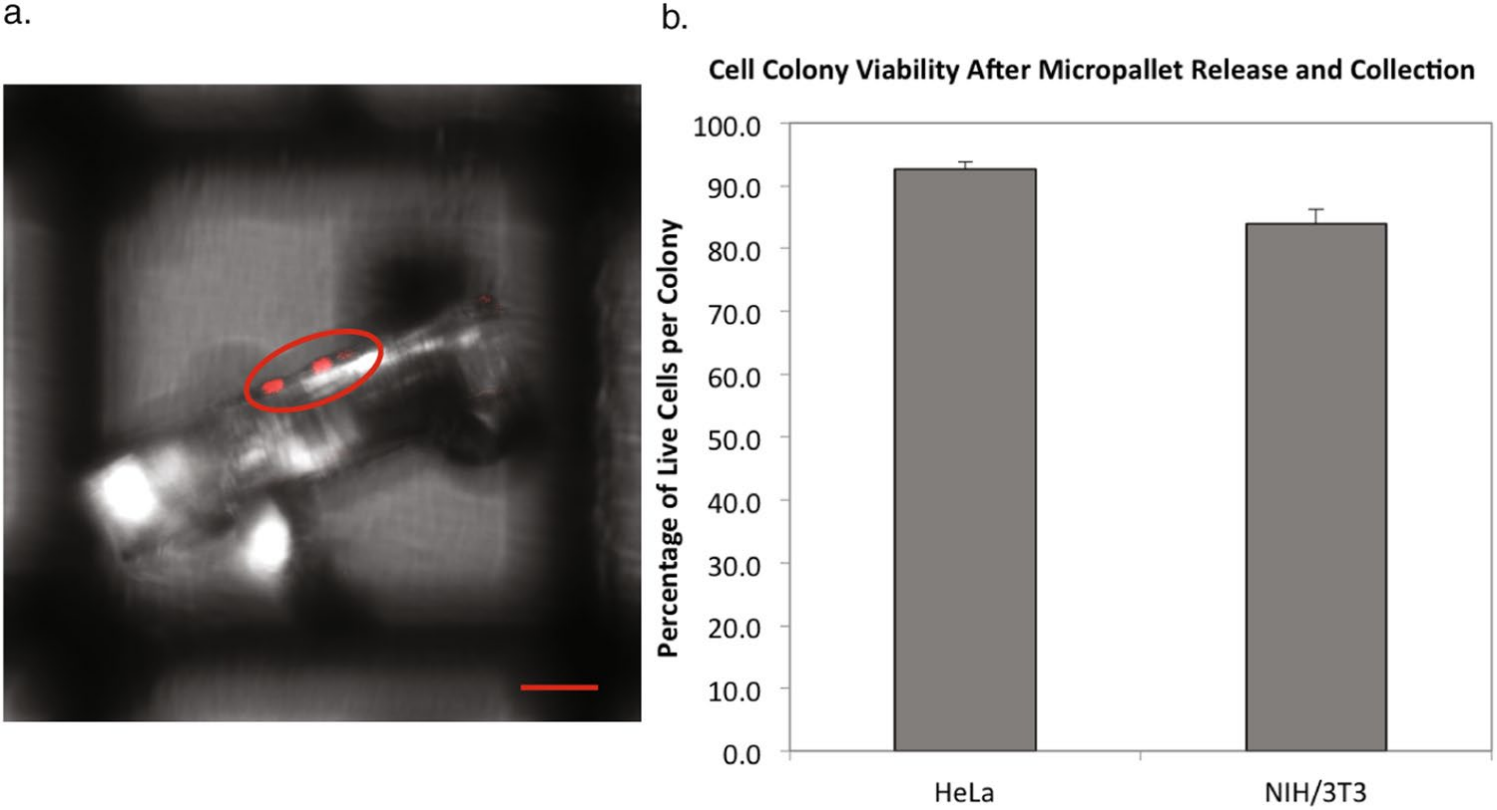
Targeted release and collection of single colonies on FCMPs. (**a**) Representative fluorescent confocal image of a released FCMP with a R208f H2b-mCherry cell colony (red pseudocolor), including a 50 μm scale bar. (**b**) HeLa and NIH/3T3 cells were seeded on separate fibronectin coated FCMPAs and grown for seven days before releasing and collecting cell colonies (n = 5) directly into Trypan blue solution. Cells were assessed for viability within each collected colony. Each FCMP carried an average of 35 ± 2.0 and 21 ± 1.4 (standard error) cells per colony.

We employed three distinct cell phenotypes; 1) wild type rat 208 f (R208f) cells (ECACC 85103116, Salisbury, UK), 2) R208f cells transfected with Jaagsiekte sheep retrovirus (JSRV) envelope (ENV) protein, previously demonstrated to be sufficient for transformation^40^, fused to a C-terminal FLAG tag epitope (JSRV-ENV-FLAG)^41^, and 3) R208f cells transfected with histone 2b (H2b)-mCherry expression plasmid^42^ resulting in nuclear fluorophore expression. Single cells from all cell types settled onto fibronectin coated FCMPs within 1 hour, adhering after 3 hours. There were no morphologic differences at low density, soon after adherence, but H2b-mCherry R208f cells could be identified by fluorescence. After seven days, during which the micropallet arrays were examined microscopically at least once a day beginning on the day of plating, the three cell types formed sequestered colonies that covered the top micropallet surfaces without exhibiting confluence (Fig. 4). At this low density, JSRV-ENV-FLAG R208f colonies contained cells with spherical morphology and could be distinguished from the other R208f colonies using confocal or phase microscopy. This provided proof of principle for a clonal isolation based on morphology, as might be employed with differentiating stem cell progeny. JSRV-ENV-FLAG transformed cells exhibited near double the growth rate of R208f wild type cells (Figure S2).

**Figure 5:**
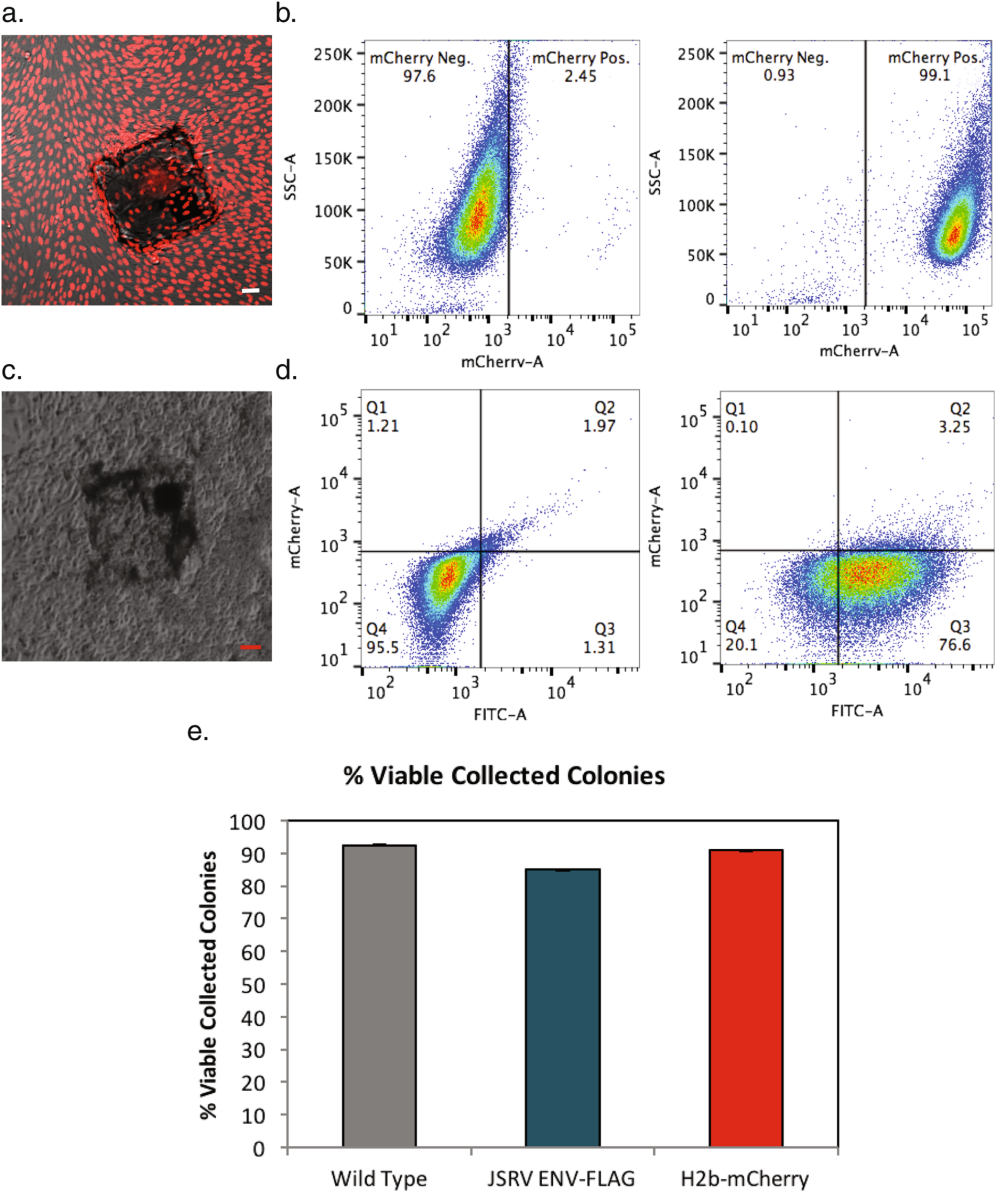
Representative analysis of individual cell colonies collected on FCMPAs that re-populated in standard cell culture wells. Representative phase contrast images containing 50 μm scale bars and flow cytometric analyses of (**a**) a H2b-mcherry rat 208 f cell population, purified from a mixture with untransfected cells, and (**b**) a JSRV ENV-FLAG rat 208 f population purified from a mixture of cells expressing H2b-mCherry. Each pure population expanded from single colonies isolated using FCMPAs. (**c**) Cell viability of wild type, JSRV ENV-FLAG and H2b-mCherry R208f cell colonies grown on fibronectin-coated micropallets. Micropallets were released and collected (n = 42, 40, and 11 for R208f WT, JSRV ENV-FLAG and H2b-mCherry respectively) with viability assessed by successful outgrowth after collection.

Two additional examples further document the capacity of this platform. Previously transfected H2b-mCherry R208f cells were noted to contain 11% fluorophore positive cells by flow cytometry, after several weeks of continuous culture under selective pressure (Fig. 5). This phenomenon is well established^43^ and often necessitates the re-purification or re-derivation of the desired clone from the phenotypically heterogeneous population. This re-derivation was rapidly accomplished with this platform. A single cell suspension of this heterogeneous population was applied to fibronectin coated FCMPAs. After single cell sequestering, the growth of clonal cell colonies, laser release, ferromagnetic recovery, and expansion culture from one colony, a pure population of H2b-mCherry R208f cells was re-established in less than 10 days (Figs 3, 5 and S1).

To unambiguously demonstrate the purity of JSRV-ENV-FLAG R208f colony sorting and the absence of cross-contamination onto adjacent micropallets with non-transformed colonies, we utilized the re-established R208f H2b-mCherry cell line and the JSRV-ENV-FLAG R208f cell line. Pure cultures of each were mixed at a ratio of 4.15% JSRV-ENV-FLAG R208f cells within H2b-mCherry R208f background (Fig. 5) and seeded onto FCMPAs as single cell suspension. By no later than 7 days of growth on the FCMPAs, pure colonies, arising from single cells of JSRV-ENV-FLAG R208f cells tracked daily by microscopic examination, could be distinguished from R208f H2b-mCherry cell colonies by their distinct morphology under light microscopy as well as absence of fluorescence. Identified morphologically transformed colonies were released, recovered, and expanded in culture. The homogeneity of the expanded population was confirmed by fluorescent microscopy and flow cytometry for the FLAG tag on JSRV ENV (completely positive) as well as the absence of cells with mCherry expression in the expanded population (Fig. 5). Thus, JSRV-transformed cells were rapidly cloned in one step from a mixture in which they represented less than 15% of the original cells.

## Discussion

We have demonstrated that the ferro-core micropallet array platform (FCMPA) rapidly and efficiently isolates pure, clonal cellular populations from heterogeneous mixtures in a single step process that can be achieved in as little time as two weeks. The fabrication and physical properties of the FCMPA have been described previously^8^, however additional refinements to sustain ECM coating of the micropallet (surface roughening) and cell sequestration were important to actualize the technology. This platform and application incorporates, the capacity for use of extracellular matrix components matched to cell type, single instrument micropallet release, and the capacity for magnetic recovery while preserving maximal optical imaging capabilities. We demonstrated rapid and efficient sorting of H2b-mCherry R208f cells from untransfected and/or non-expressing R208f cells remaining in the transfection mixture, and separate purification of JSRV ENV-FLAG R208f cells^44^ within a H2b-mCherry R208f cell background. Cell colonies grown on ferro-core micropallets can be magnetically recovered after laser release, in a high throughput capacity, with excellent cell viability. Complete purification of transfected cells from low concentrations within heterogeneous mixtures could be achieved in as little time as two weeks using ferro-core micropallet arrays. The FCMPA provides a novel tool to allow investigators to more rapidly generate critical reagents to address a multitude of fundamental biological questions.

The ferro-core micropallet array platform has distinct advantages over currently existing technologies and methods. Fluorescence activated cell sorting (FACS) has at least five discrete limitations^5^ delineated earlier in the Introduction. Similarly, isolation of clonal populations of adherent cells by limiting dilution or cloning rings^6,7^ either requires screening of a large number of culture wells for colony growth and phenotyping or repetitive application of small rings to encircle and selectively harvest the desired adherent cell colonies^6^, a laborious and time-consuming process. These methods remain essentially unchanged since their original description decades in the past. The use of cloning rings, as with FACS, rarely results in the isolation of a pure population of cells, often representing a mixture of transfected and non-transfected cells, desired and non-desired cells.

Other microstructure platforms have been described^16,28,29,45^. Each of these has particular challenges for widespread application in biological studies. Earlier iterations of the micropallet array platform employed separate imaging and laser release instrumentation along with an inversion recovery system that results in low throughput recovery of released micropallets^12–14,16,20^. Although the former issue has been resolved with development of methods to use the laser of the scanning confocal microscope to release the micropallets^9^, the recovery of selected and released non-magnetic microstructures remains problematic. Initial efforts to improve recovery of released micropallets incorporated ferromagnetic nanoparticles into the matrix of the microstructures, which achieved the desired capacity to employ magnetic recovery for the micropallets and adherent cells but, at a cost of significant degradation of imaging capabilities^9,23,32^.

The issue of potential cross contamination remains a concern for platforms that rely upon cell growth across microstructures, especially when using fibronectin as the extracellular matrix coating given its propensity to polymerize into sheets across the entire micropallet array surface^19^. Increased spacing, physical separation, of larger micropallets can mitigate this concern, but obviates the use of virtual air walls^20,25,28,29,45^. The microraft platform has been proposed as a solution to this challenge, however to date the designed cell growth surface has been limited to polystyrene or a copolymer mixture of polylactic acid and polyglycolic acid^30,31^. This limits the flexibility of the platform to accommodate the extracellular matrix preferences of primary cells and cell lines. Additionally, the micro-raft platform employs physical walls of PDMS to constrain the growth of cells to a limited surface. Although, PDMS is not as supportive of adherent cell attachment as growth structures coated with extracellular matrix components, PDMS does support adherent cell attachment and growth^46–48^, raising concerns about cross microraft contamination. Our decision to incorporate perfluorotributylamine, Fluorinert FC-43, in lieu of virtual air walls or physical PDMS walls mitigates this concern, as the characteristics of this liquid preclude cell adhesion in the absence of surfactant or a deposited protein layer, neither of which are incorporated into this refined FCMPA platform. An additional advantage to this selection, is the removal of the time consuming silanization procedure from the fabrication process, making the fabrication of these FCMPAs less time consuming than any of the previously described micropallet or microraft platforms for cell colony isolation.

We employed standard plasmid transfection of a fluorescent reporter fusion protein construct, requiring antibiotic selection, as well as a plasmid expressing a transforming protein from an oncogenic retrovirus^41,44^. We demonstrated efficient sorting of H2b-mCherry R208f cells from untransfected and/or non-expressing R208f cells remaining in the transfection mixture, and separate purification of JSRV ENV-FLAG R208f cells^44^ within a H2b-mCherry R208f cell background. Cell colonies grown on ferro-core micropallets can be magnetically recovered after laser release, in a high throughput capacity, with maintained cell viability.

Complete purification of transfected cells from low concentrations within heterogeneous mixtures could be achieved in as little time as two weeks using ferro-core micropallet arrays. This time frame is demonstrated by our experiments with established cell lines in which cells are applied and grown to cell colonies on a ferro-core micropallet array for seven days thereafter, sequestered cell colonies grown on ferro-core micropallets were collected and transferred to 96-well plates for seven days of continued growth. We recognize that the transformation process and selection process for these cell lines exceeded this two-week time frame. However, with efficient transduction, non-toxic reporter function, and robust cells, this two-week time frame is achievable and would be so in the case re-derivation of a desired clonal population. The procedure only required a single microcolony collection event and was much faster than typical purification procedures that can take several months to complete. Single cell sequestering to individual micropallets allowed single attached cells ample area to grow without contact to other nearby cells on adjacent micropallets. The discontinuous micropallet array surface eliminated issues associated with varied cell growth characteristics among transfected and untransfected cells in culture, ensuring that slow growing transfected cells could be purified from populations consisting of more rapidly growing cell populations.

As with any technique, there are limitations to the successful application of the ferro-core micropallet array. Although a fibronectin ECM coating was used for demonstration of the ferro-core micropallet array workflow, we have reported methods for coating micropallets with other ECM coatings, such as laminin-5, collagen, and basement membrane extract^19^. Evidence of cell lines exhibiting preferential adherence towards different ECMs^19,49^ suggests that specific ECM coatings may be best suited for the ferro-core micropallet array and should be tailored to the cell type of interest. Although we used R208f cells of three different phenotypes for this work, the successful use of other cell lines on micropallet arrays including 3T3, Hela, COS, has been well documented and more recently primary tumor cells (data not shown). This platform does not permit partial sampling of the micro-colony. However, we would argue that traditional cloning methods also do not readily accommodate this when limited numbers of cells reside within the presumed clonal population. Given the extensive development of non-lethal reporter constructs and increasing use of fluorophore labeled aptamers and oligonucleotide probes as intracellular probes in intact and viable cells^50–56^, the necessity of cell lethal screening modalities is not as critical as in past generations of molecular biology studies.

We acknowledge that there is increasing concern regarding cell line identity and that within cell lines maintained in culture there can be genetic drift or cross contamination. Thus, in some instances phenotypic characterization may need to be followed by genotypic verification of a cell line’s construct and by extension its clonality. Never the less, as a matter of common practice, initial phenotypic screening is the first step in establishing a verified clonal cell population. The ability to visualize the ferrocore micropallet array with a standard or phase contrast microscope in a longitudinal fashion along with the fact that we have no evidence that cells cross the Fluorinert, physical boundary, occupying the “streets” between micropallets permits the rapid isolate a phenotypically pure microcolony from a single cell. We have demonstrated this capacity which, strongly supports the utility of this platform to greatly accelerate this mainstay of molecular and cellular biology.

The ferro-core micropallet array technology has applicability beyond cellular transfections and can be broadly applied towards enhancing adherent cell colony sorting. The performance characteristics of the FCMPA and this methodology compare very favorably with data derived from other micropallet and microraft platforms. The flexibility of imaging modality/parameters and of matching extracellular matrix component coatings to the cell type of interest provide additional benefits over and above the reliable cell sequestration, ease of micropallet release, efficient magnetic recovery while avoiding the degradation of image quality that limits multichannel imaging. The ferro-core micropallet array provides a reliable and efficient platform to rapidly isolate clonal cell populations from heterogenous cellular mixtures and allows investigators to more rapidly address a multitude of fundamental biological questions involving heterogeneous, normal, and pathological primary tissues dominated by adherent cells.

## Methods

All chemicals, cell culture media, media supplements, disposables, and reagents were procured from Fisher Scientific (Pittsburgh, PA) unless otherwise noted.

### Ferro-core micropallet array fabrication

Ferro-core micropallet arrays (FCMPAs) were photolithographically fabricated onto the surface of thin gold coated glass microscope slides and electroplated with ferro-magnetic material, as described previously^8^ and are available commercially (Celligi Inc. Irvine, CA). Briefly, glass slides were coated with a 20 Å titanium seed layer followed by a 200 Å gold layer by electron beam vapor deposition. Subsequently, overlying arrays consisting of micropallets fabricated from 1002 F photoresist^8,34^ with dimensions of 250 µm (w) by 250 µm (l) by 50 µm (h) and 50 µm gaps between adjacent micropallets, with indexing notches, 30 µm × 30 µm square regions, incorporated into the edges of the micropallets, in sections of five by five micropallets, patterned with one of nine different marker designs. To enable magnetic retrieval of the micropallets, a single 60 µm (w) by 60 µm (l) hole was included within each micropallet spaced 30 µm from a single corner to be filled with an electroformed ferromagnetic core. This core consisted of three layers, as described previously^8^.

### Ferro-core micropallet array post-fabrication treatment

To enhance extracellular matrix protein and cell adhesion to the pallets, the magnetic micropallet array surfaces were roughened using a procedure adapted from a previously documented method^16^ utilizing a slurry of 1 µm aluminum oxide (Al_2_O_3_) particles (Sigma Aldrich, St. Louis, MO) mixed in double distilled water (ddH_2_O) at a 1:1 ratio, to superficially abrade the top surface of the micropallets. Once the surface roughening procedure was complete, the arrays were washed three times with alternating immersions into ddH_2_O and 70% ethanol removing all excess Al_2_O_3_ particulates. LabTek culture chambers (Nunc, Naperville, IL) were adhered over the surface of the ferro-core micropallet arrays with cured polydimethyl siloxane (PDMS) to constrain liquid reagents. Both 1000 µL (4-well) and 500 µL (8-well) capacity chambers were used. The completed FCMPAs were sterilized with 70% ethanol and kept in a sterile environment until use.

Sterile FCMPAs were prepared for use with human fibronectin protein (huFN, Millipore, Billerica, MA) deposited onto the top surfaces of FCMPs to facilitate cell adhesion as previously described^19^. To sequester cells on the top surfaces of the micropallets, liquid wall formation between micropallets was established just before cell seeding. Fluorinert FC43 (3 M, Saint Paul, MN), a fluorinated liquid with a liquid density of 1860 kg/m^3^ was used to form barriers in between individual pallets. For 4 and 8 well Lab-tek chambers, 1.0 and 0.5 µL of FC43 was placed in the middle of each culture well, respectively, and given 5 seconds to spread through the pallet gaps. A volume of 500 µL fresh culture media was then immediately added gently to the wells in order to prevent evaporation of the volatile FC43 and the arrays were allowed to settle for 5 minutes before the addition of the desired number of cells. The high density of the FC43 effectively forced the liquid to the bottoms of the wells when mixed with the less dense culture media, forming barriers between each pallet.

### Cellular Reagents

FLAG-tagged Jaagsiekte sheep retrovirus envelope expression plasmid (JSRV-ENV-FLAG)^40^ and mCherry-tagged histone 2b fluorescent expression plasmid (H2b-mCherry) a gift from Robert Benezra (Addgene plasmid #20972)^42^ were transformed into competent *E. coli* DH5α for propagation of the plasmid using established protocols^40^ and standard heat shock transfection procedures under ampicillin, 100 μg/mL, selection pressure. Plasmid DNA from overnight cultures derived from a single bacterial colony was isolated using a Qiafilter midiprep plasmid purification kit (Qiagen, Valencia, CA) according to manufacturer’s instructions and quantified using a nanodrop ND-1000 spectrophotometer (ThermoScientific, Waltham, MA). The JSRV-ENV-FLAG plasmid was confirmed using restriction enzyme (*NcoI*, 2.1 and 4.1Kb bands) digestion while the H2b-mCherry plasmid was confirmed using two digestions (*AatII*, 5.78, 0.37, 0.19, 0.08, and 0.05Kb; *XbaI*, 6.48 Kb) with DNA fragments visualized via standard agarose gel electrophoresis including appropriate size control ladders. The purified plasmid DNA was stored at −20 °C until cellular transfections.

Rat 208 f fibroblasts (R208f), (European Collection of Authenticated Cell Cultures [ECACC] Salsbury, UK) used for transfection were described previously^40^ and cultured per ECACC recommendations. Wild type or transfected R208f cells were released from their culture dishes by incubation with 0.25% Trypsin and 0.1% EDTA solution in 1X PBS, pH 7.4, washed with cell culture media and collected by centrifugation at 228 × g at room temperature for 5 minutes. The cell pellets were resuspended in media or buffer and counted on a hemocytometer using Trypan Blue exclusion for the identification of live cells. R208f cells at 100,000 cells/well were seeded in a 6-well dish in standard media (2 mL/well) for plasmid transfections that were achieved using Fugene6 (Promega, Madison, WI), according to manufacturer’s instructions. A total of 4 μg of JSRV-ENV-FLAG or 2.5 μg of H2b-mCherry plasmid DNA was mixed into a mixture of 16 μL Fugene6 and 200 μL of OptiMEM media (Lifetechnologies, Carlsbad, CA) incubated for 15 minutes at room temperature before being transferred to the appropriate well of a cell culture 6-well dish for 24 hours when it was replaced with cell culture media containing penicillin and streptomycin. Forty-eight hours post-transfection, 600 μg/mL G418 antibiotic was supplemented into the cell culture media of R208f cells transfected with H2b-mCherry for positive selection. JSRV-ENV-FLAG transfected R208f cells did not require antibiotic selection.

### Cell clone and cell population characterizations

JSRV-ENV-FLAG transduced R208f cells were detected by morphological signs of oncogenic transformation and/or by immunofluorescent staining of the encoded FLAG tag, which required cell permeabilization. An equal volume of 4% paraformaldehyde solution was added to the sample to fix the cells for 20 minutes. Samples were then washed twice with 1X permeabilization buffer (Biolegend, La Jolla, CA), maintained in this buffer for the remainder of the staining procedure, and stained with anti-FLAG mouse monoclonal antibody (Cell Signaling, Danvers, MA) or Mouse IgG1, K isotype control antibody (Biolegend, La Jolla, CA) at 0.24 μg/mL for 30 minutes and washed twice. FITC labeled goat anti-mouse IgG (H + L) secondary antibody (Jackson Immunoresearch, West Grove, PA) was then applied to the samples at 13.0 μg/mL for 30 minutes at room temperature then washed twice with 1X permeabilization buffer. A BD LSR II flow cytometer equipped with 488 nm and 561 nm excitation lasers and appropriate FITC (525/50 nm) and mCherry (610/20 nm) bandpass filters was utilized for all flow cytometry. H2b-mCherry transfected R208f were visualized by confocal microscopy on a Zeiss LSM 780 with a paired two-photon laser system (Mai Tai, Spectra-Physics) using a 561 nm laser (6% power) for excitation and with a detection channel spanning 590–640 nm.

For all flow cytometry experiments, a total of 50,000 single cell events per sample were collected for analysis by first creating a FSC-A vs. SSC-A gate to exclude debris at the origin; single cell events were gated by FSC-A vs. FSC-H to exclude cellular aggregates. H2b-mCherry and FITC detection gates were set using an appropriate Mouse IgG1, K isotype control or un-transfected R208f cells accordingly. Dot plots contain 50,000 single cell events. Acquiring fluorescence data of both FITC and mCherry did not require compensation on the BD LSRII. Data analysis was performed using FlowJo software version 10.1 (TreeStar, Ashland, OR).

All cell types were prepared in the same manner for seeding to the ferro-core micropallet arrays. To achieve single cell sequestering to individual FCMPs, the total number of cells seeded per single Lab-tek well was equal to one third the total number of micropallets contained within a single well as defined by a random Poisson distribution, i.e. 250 cells were seeded into 8-well chambers and 500 cells were seeded into 4-well chambers. Cells that were seeded onto the arrays were grown for seven days with media exchanges at 72 hours and 144 hours. Cell growth rates were determined by daily manual counts of cell number on consistently identified micropallets.

Colonies of interest were specified as those that were exhibiting the desired features of morphology or mCherry fluorescence, as defined for the specific cell type. Once a desired colony was located, the micropallet bearing the colony was ejected using the two photon laser as previously described^8^. Briefly, the confocal microscope was set up for dual photon exposure, focused at the gold film layer beneath a target micropallet, and set to excite at 790 nm. The pallet was typically released with an average total applied energy of 88mJ. Once released, the FCMPs were magnetically collected with a probe with a removable magnet in a similar manner to that previously described^9^ and transferred to a multi-well culture dish prepared with fresh culture media for continued growth.

## Electronic supplementary material

Supplemental Information
