# Supplementary material for: Highly efficient cellular cloning using Ferro-core Micropallet Arrays
Source: Sci Rep. 2017 Oct 12;7:13081. doi: 10.1038/s41598-017-13242-1 (PMC5638909; doi:10.1038/s41598-017-13242-1)
Supplement: Supplementary file 1 — Supplemental Information [file 41598_2017_13242_MOESM1_ESM.pdf]

Highly efficient cellular cloning using Ferro-core Micropallet Arrays

Trisha M. Westerhof[a,e], Wesley A. Cox-Muranami[b,e], Guann-Pyng Li[b], Mark Bachman[b], Hung Fan[c,d], and Edward L. Nelson[a,c,d,*]

[a]School of Medicine, Department of Medicine, Division of Hematology/Oncology, University of California, Irvine.

[b]Samueli School of Engineering, Department of Biomedical Engineering, University of California, Irvine.

[c]Francisco Ayala School of Biological Sciences, Department of Molecular Biology and Biochemistry, University of California, Irvine.

[d]NCI-designated Chao Family Comprehensive Cancer Center.

[e]The authors contributed equally to this work.

[*]**Correspondence to:** Edward L. Nelson, 839 Medical Sciences Ct. Sprague Hall Room 136, University of California at Irvine, Irvine, CA 92697. Phone: 949.824.2860, Fax: 714.456.2242, email: enelson@uci.edu.

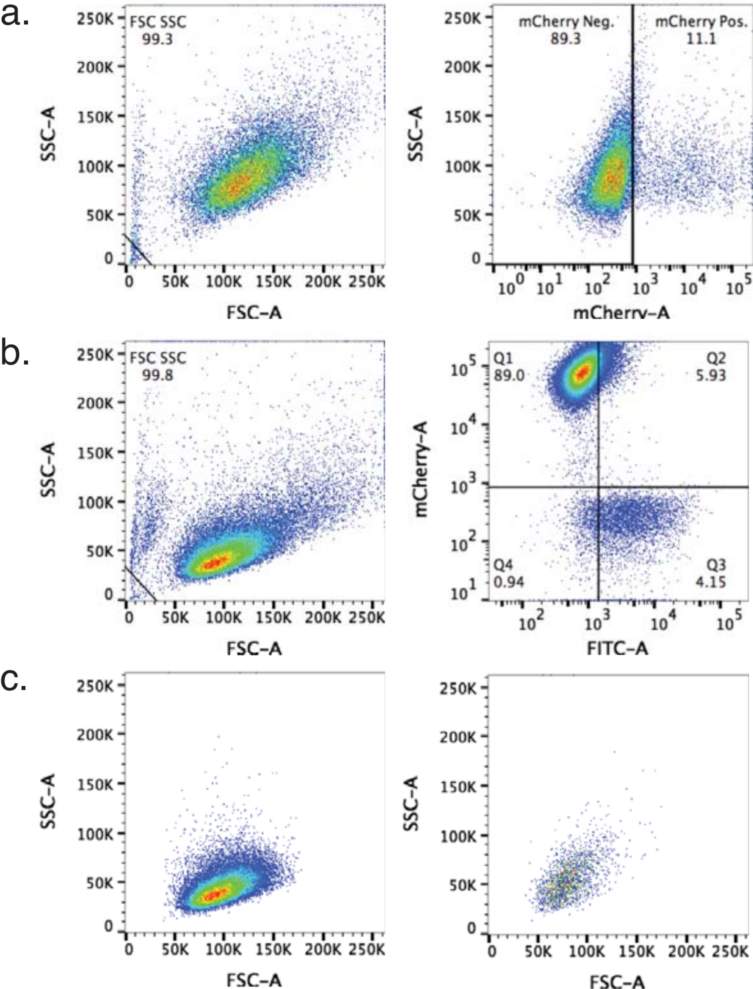
a.
FSC SSC
99.3
SSC-A
FSC-A
250K
200K
150K
100K
50K
0
50K
100K
150K
200K
250K
mCherry Neg.
89.3
mCherry Pos.
11.1
mCherry-A
10^0 10^1 10^2 10^3 10^4 10^5
b.
FSC SSC
99.8
Q1
89.0
Q2
5.93
Q3
4.15
Q4
0.94
mCherry-A
FITC-A
10^1 10^2 10^3 10^4 10^5
c.
SSC-A
FSC-A

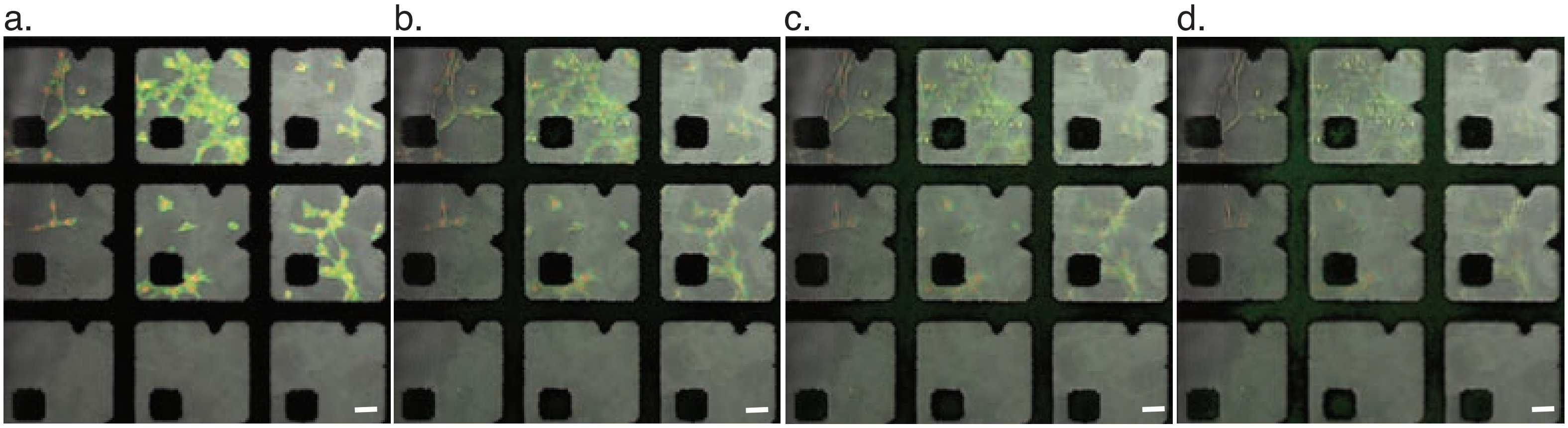
a.
b.
c.
d.
